# Lead and mercury exposure in pregnant women in the UK: a cross-sectional observational study (the PEAR Study)

**DOI:** 10.1136/bmjopen-2025-114169

**Published:** 2026-06-29

**Authors:** Caroline M Taylor, Linda Mottram, Jenny Ingram, Jane Entwistle, Katarzyna Kordas, Bruce Lanphear, Jean Golding, Jackie Morton

**Affiliations:** 1 Centre for Academic Child Health, Bristol Medical School, University of Bristol, Bristol, UK; 2 Geography and Natural Sciences, Northumbria University, Newcastle upon Tyne, UK; 3 Department of Epidemiology and Environmental Health, School of Public Health and Health Professions, University at Buffalo, Buffalo, New York, USA; 4 Simon Fraser University, Burnaby, British Columbia, Canada; 5 HSE Science and Research Centre, Buxton, UK

**Keywords:** PEAR, toxic metal, pregnancy, women, lead, mercury

## Abstract

**Abstract:**

**Objectives:**

Lead and mercury are recognised as critical determinants of population health, especially for children and pregnant women. Few UK cohorts have measured lead or mercury in pregnant women since 1990. The aims of this study are to: (1) quantify lead and mercury exposures in a sample of pregnant women in the UK; (2) document concentrations over time in pregnant women in the UK; and (3) compare concentrations with international data.

**Design:**

Whole blood samples were obtained from pregnant women in a quantitative arm of the Pregnancy, the Environment And nutRition Study in a cross-sectional observational study design. The samples were analysed by inductively coupled plasma mass spectrometry (Health and Safety Executive, Buxton, UK).

**Setting:**

Community-based with recruitment through a hospital antenatal clinic in south-west England.

**Participants:**

Women ≥18 years old and ≥11 weeks pregnant by last menstrual period.

**Primary outcome measures:**

Whole blood lead and speciated mercury concentrations.

**Results:**

The whole blood lead concentration was: mean 5.8 (SD 6.2), median 4.6 (IQR 3.5, 5.9), range 1.7–78.1 µg/L (n=262; gestational age range by scan 8.3–15.9 weeks). These values are about 84% lower than in pregnant women in the UK Avon Longitudinal Study of Parents and Children (ALSPAC; 1991–1992), about 47% lower than in the UK Born in Bradford (BiB) study (2007–2011) and about 34% lower than in White women in the UK Mother’s and Baby’s Exposure to Lead study (MaBEL; 2011). Blood total mercury concentration was: mean 0.83 (SD 0.64), median 0.69 (IQR 0.34, 1.19), range 0.07–3.40 (about 60% lower than in pregnant women in ALSPAC and about 37% lower than in BiB). These results are similar to contemporary concentrations found in Europe and the USA.

**Conclusions:**

Blood concentrations of lead and mercury in pregnant women in the UK have declined considerably since the early 1990s. However, no safe levels have previously been identified for adverse effects on cognition, preterm birth rate or coronary heart disease prevalence. These data will contribute to the ongoing development of public health advice.

**Study registration:**

ISTCTN92638336.

**STRENGTHS AND LIMITATIONS OF THIS STUDY**

- The study provides a valuable contemporary measurement of toxic metal exposures (mercury and lead in the same group of women) in a vulnerable group in the UK, significantly expanding and updating a very small body of data from the UK.
- All the women were at a similar gestational age (<16 weeks), largely avoiding variation in the blood volume expansion in the second trimester.
- We have included data on speciation of blood mercury, which indicates sources of exposure and for which there are no previous UK data for pregnancy.
- The findings are not generalisable to other areas of the UK, where exposures may be different due to, for example, variations in the density of industry and the metallic content of rocks and soil, or in the amount of lead-shot game consumed.
- Similarly, the results are not generalisable to populations with a different demographic profile (particularly regarding the limited representation of diverse ethnic groups and relatively high socioeconomic status).

## Introduction

 Exposure to lead and mercury is a critical concern for population health, especially for vulnerable groups such as children and pregnant women. Lead and mercury exposures are recognised by the WHO as a threat to the development of children and are among the top ten chemicals of major public health concern.^1^

Lead and mercury pass freely through the placenta^2 3^ and have neurotoxic effects on the fetus, and/or cause epigenetic changes^4^ that may affect the future development of the child. During pregnancy, toxic metals may contribute to adverse outcomes including pre-eclampsia, preterm delivery and babies that are small for gestational age.^1 5^ The fetus is particularly vulnerable to the effects of toxic metals because of the high rate of cell division and differentiation, with lifelong consequences. In utero exposure to lead is adversely associated with child IQ, with impact on individual developmental and economic life course trajectories, as well as financial and societal costs at a population level.^6^ Mercury also has adverse neurodevelopmental effects, including associations with learning and memory deficits, delayed cognitive performance, preterm birth and birth defects.^7^

For toxic metals such as lead and mercury that have any degree of transfer through the placenta to the fetus, no identified ‘safe’ limits for maternal levels have been identified.^8^ The only means of reducing fetal exposure is to minimise maternal exposure. Pregnant women receive wide-ranging health-related information to optimise the health and the development of the fetus. Of relevance to lead and mercury exposure in the UK is guidance on foods to avoid or limit, which is available on the National Health Service (NHS) for website for England^9^: this includes guidance on fish consumption (mercury) and on lead-shot game meat/gamebirds (lead). This guidance is especially important as food and drink are now among the main sources of exposure to toxic metals in Europe.^10^,^12^

There are important gaps in knowledge on which the UK NHS guidance for foods to avoid or limit in pregnancy is based. These include a lack of data on current exposure levels in pregnancy in the UK. There are to our knowledge only three cohort studies since 1990 that include information on toxic metal exposures in pregnancy in the UK: (1) the Avon Longitudinal Study of Parents and Children (ALSPAC), which includes prenatal measures of blood lead and total mercury (1991–1992)^13^; (2) the Born in Bradford (BiB) study, which includes prenatal measures of whole blood lead and total mercury (2007–2011)^14^ and (3) the Mother’s and Baby’s Exposure to Lead (MaBEL) study, which includes prenatal measures of whole blood lead in South Asian and White women in Leeds (2011).^15^ In addition, a study by Health Survey for England included blood lead concentrations in women of childbearing age in 1995 (not specifically pregnant) in a nationally representative sample.^16^,^18^ Although exposure to toxic metals from industrial emissions has declined, at least for lead and mercury, current exposure levels of pregnant women to these metals in the UK are unknown. There are no data to our knowledge on exposure to methylmercury (MeHg), which is the most toxic form of the metal and is biomagnified in aquatic species including fish, and may have an increased rate of placental transfer compared with inorganic mercury.^19 20^ Important sources of lead and mercury remain in the environment, and there is increasing recognition that there are no ‘safe levels’ of exposure below which there may be no adverse effects.^8^

The aims of this study are as follows: (1) to determine exposure levels to lead (blood and urine), mercury (both speciated and total levels in blood and in total levels in urine) in a sample of pregnant women in Bristol, UK; (2) to compare exposures in pregnancy and in the wider population in the UK where data exist; and (3) to compare exposure data in the study sample to levels observed internationally and to established levels of concern where these exist. These findings will be of value in informing: (1) current guidance on public health policies and information in pregnancy; and (2) the need for screening and individual advice and treatment.

## Methods

### Participants and recruitment 

Women were eligible for the study if they were: (1) ≥11 weeks gestation by last menstrual period dating; (2) ≥18 years old; (3) booked for antenatal care at Southmead Hospital, North Bristol Hospital Trust, Bristol, UK; and (4) intending to attend the 12-week antenatal booking clinic and intending to provide a blood sample as part of their routine expected care. This recruitment was independent of the sample of postpartum women recruited for a qualitative arm of the study.^21 22^ Potential participants were identified from the clinic list for the 12-week hospital antenatal clinic. The study Research Midwife contacted potential participants by phone: if they expressed interest and agreed, they were sent a participant information sheet (including a link to the study website (https://pearstudy.com/) and contact details for further information) and a consent form by email. Participants who did not return the completed consent forms were not contacted further.

### Data collection 

Data collection took place in 2023–2024. On receipt of the completed consent form, each participant was asked to complete an electronic screening questionnaire (T0) to confirm eligibility and the date of their booking clinic appointment. The routine care at the antenatal clinic included an ultrasound scan and a venous blood sample taken by a midwife assistant.

Participants who completed the baseline questionnaire T0 were asked to complete a further electronic questionnaire (T1). This included information about: (1) demographic variables including for example household income, educational attainment and ethnicity; (2) potential sources of exposures to lead and mercury, such as smoking or vaping, employment and hobbies, water sources, age of housing and household renovations; (3) emesis and cravings/pica behaviour during the pregnancy; (4) dietary preferences such as being a vegetarian; (5) consumption frequency of specific food and drink items, including those related to toxic metal exposures and those included in the NHS guidance on items to avoid or limit during pregnancy relevant to toxic metal exposure (eg, fish, lead-shot gamebirds and game meat) and (6) knowledge of advice on healthy eating and on foods/drinks to avoid or limit sources during pregnancy and sources of information for these guidelines.

### Biosamples

Following consent, participants were mailed: (1) a urine sample home collection kit comprising a 10 mL universal container and (2) a 5 mL EDTA blood collection tube to take to the 12-week booking clinic. The kit also included postal-approved packaging for the tubes and prepaid envelopes for return of the samples directly to the laboratory. To maximise the sample collection rate, a phone text reminder to take the blood tube to the clinic was sent the day before their appointment and the clinic staff had a list of participants with appointments on any specific day. Spare blood tubes were available at the clinic if the participant forgot to bring their tube to the clinic. Reminders to return the urine collection tube to the laboratory were sent as text messages at 2 and 3 weeks after posting if the sample had not been received by the laboratory. The date of collection was noted to enable derivation of gestational age at sampling. Samples were batched on receipt at the laboratory; urine samples were stored frozen at −20°C and whole blood samples were refrigerated at 5°C until analysis.

### Data management

Data were managed using REDCap software.^23 24^ This was used as the host platform for consent forms and questionnaires and to record information on samples returned. The telephone messaging service Twilio^25^ was linked to REDCap to send text messages including reminders to the participants.

### Withdrawals

Withdrawal of consent was communicated through direct contact from the participant or from the hospital Research Midwives. Participants were informed that their data provided up to point of withdrawal would be retained and used in analyses unless they informed us that they did not wish their data to be used.

### Analytical methods for blood and urine analyses

Samples were analysed at the Health and Safety Executive, Buxton, UK by inductively coupled plasma mass spectrometry (ICP-MS) for total metals and by liquid chromatography-ICP-MS for the determination of individual mercury species. Analytical methods are described in detail in online supplemental text. Samples that were below the limit of quantification (LOQ) were assigned a concentration equal to LOQ/√2 (see online supplemental tables S1 and S2).^26^

### Statistical analyses

Statistical analysis was done using SPSS V.30.0.0.0 (172) using all available data. Summary statistics (mean and SD, median and IQR, range) were calculated for lead and mercury values. Demographic characteristics were described for those who completed the questionnaire and who also provided a urine sample, and correspondingly for those who completed the questionnaire and provided a blood sample. The associations of blood lead and mercury with demographic characteristics were investigated with analysis of variance (ANOVA).

## Results

**Figure 1:**
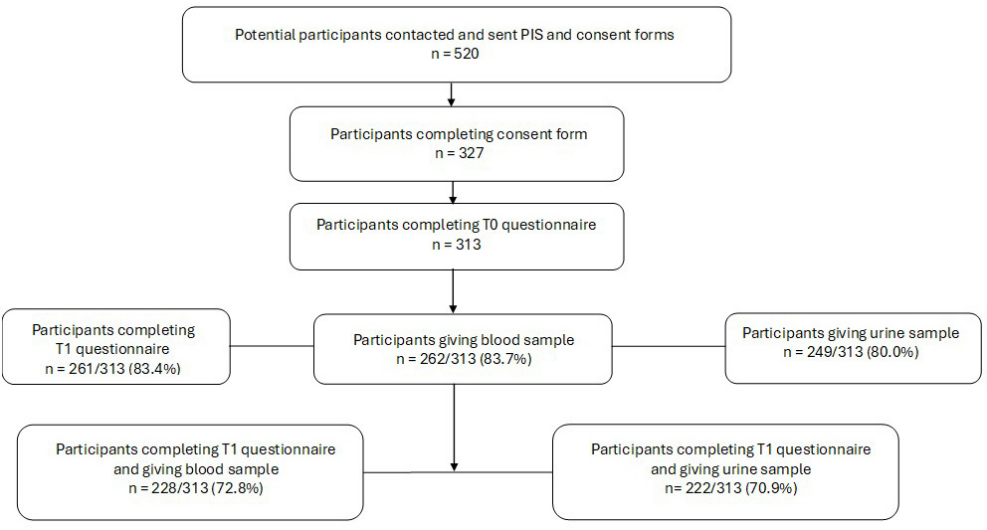
PEAR Study flow chart. n=242 participants provided both a blood and a urine sample. PIS, participant information sheet.

520 women responded to telephone contact requesting the participant information sheet and consent form. Of these, 313 (60%) pregnant women enrolled in the study (completed the baseline eligibility questionnaire (T0) and the consent form); 262 provided a blood sample and 249 a urine sample. Of those that completed the T1 questionnaire (n=261), 222 (85%) supplied a urine sample and 228 (87%) a blood sample (see flow chart in figure 1). n=242 provided both a blood sample and a urine sample. Almost 90% of the participants were of white ethnicity, most (>60%) had an annual household income >£50 000 and most had a university degree or similar educational qualification (>70%) (table 1). Few participants smoked cigarettes (<2%) or vaped (<3%).

**Table 1:** Demographic characteristics of PEAR Study participants

Characteristic	Participants with T1 and urine sample, n (%) or mean (SD) (max. n=222)	Participants with T1 and blood sample, n (%) or mean (SD) (max. n=228)
Age (years)		
<25	6 (2.7)	6 (2.7)
25–35	136 (61.8)	143 (63.3)
>35	78 (35.5)	77 (34.1)
BMI classification*		
Underweight (<18.5 kg/m^2^)	8 (6.2)	8 (6.1)
Normal weight (18.5–24.9 kg/m^2^)	66 (50.8)	68 (51.5)
Overweight/obese (≥25 kg/m^2^)	56 (43.1)	56 (42.4)
Parity		
0	119 (53.6)	122 (53.7)
≥1	103 (46.4)	105 (46.3)
Gestation age at T1 (weeks):		
<12	126 (57.8)	130 (57.8)
12–14	86 (39.4)	88 (39.1)
>14	6 (2.8)	7 (3.1)
Gestational age at sampling (weeks)	12.5 (SD 1.1), range 8.6–17.4	12.2 (SD 0.8), range 7.7–15.9
Ethnicity		
White	194 (87.8)	198 (87.6)
Black/African/Caribbean/Black British/Asian/Asian British/Mixed and multiple ethnic groups	27 (12.2)	28 (12.4)
Household income		
<£20 000	12 (5.6)	11 (5.0)
£20 000 to <£50 000	58 (27.2)	61 (32.9)
≥£50 000	143 (67.1)	147 (67.1)
Highest educational qualification		
None/GCSE/vocational level 1 and 2/AS or A level/Vocational level 3	52 (23.5)	53 (23.3)
University degree (BSc, BA, MA)/professional qualification/vocational levels 4 and 5/university higher degree (MSc, PhD)	169 (76.5)	174 (76.7)
Smoker (tobacco)		
No, not at all	219 (98.6)	225 (98.7)
Yes, less often than every day/yes, every day	3 (1.4)	3 (1.3)
Vaping*		
No, not at all	173 (97.2)	178 (97.8)
Yes, less often than every day/yes, every day	5 (2.8)	3 (2.2)
Type of housing		
Detached house (two storeys or more)/detached house (bungalow)	42 (19.1)	41 (18.2)
Semi-detached house (two storeys or more)/semidetached house (bungalow)	87 (39.5)	95 (42.2)
Terraced house/townhouse (two storeys or more)	60 (27.3)	59 (26.2)
Flat or apartment/Maisonette or duplex/other	31 (14.1)	30 (13.3)
Special diet		
Yes	33 (15.1)	31 (13.8)
No	186 (84.9)	194 (86.2)
Vegetarian no fish/vegan	14 (6.3)	13 (5.7)
Vegetarian with fish	10 (4.5)	10 (4.3)

* Question added to questionnaire part way through data collection.

BMI, body mass index; GCSE, General Certificate of Secondary Education; PEAR, Pregnancy, the Environment And nutRition.

The gestational age by scan at blood sampling was 8.3–15.9 weeks, at urine sampling 8.6–17.5 weeks, and at T1 completion 8.0–16.6 weeks.

No blood samples had a lead concentration <LOQ. 22 blood samples had total mercury concentrations <LOQ and were assigned the value 0.070 µg/L; 16 urine samples were <LOQ for lead and were assigned the value 0.050 µg/L. 11 blood samples were <LOQ for MeHg and were assigned the value 0.141 µg/L; 210 samples were <LOQ for Hg^2+^ and were assigned the value 0.141 µg/L (see online supplemental table S2).

**Figure 2:**
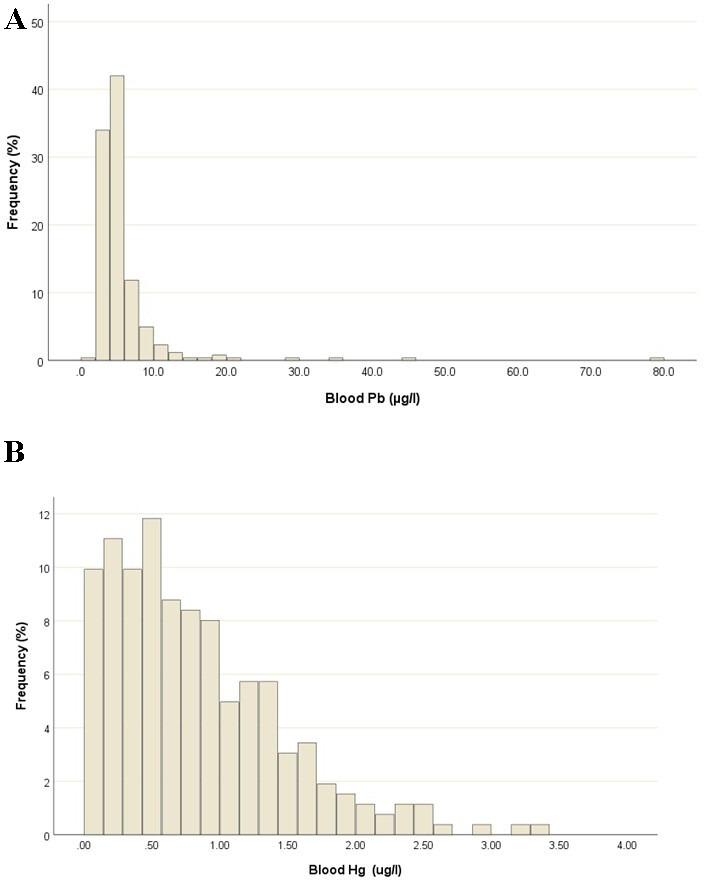
Histograms of whole blood (**a**) lead (skewness 7.66 (SE 0.15), kurtosis 76.13 (SE 0.30)) and (**b**) total mercury (skewness 1.20 (SE 0.15), kurtosis 1.55 (SE 0.30)) values in PEAR Study participants (n=262).

Blood lead concentrations (n=262) and urine lead concentrations (expressed as μg/L and μg/g creatinine; n=249) are shown in table 2 and figure 2. The data were left skewed as was also the case in ALSPAC.^27^ There was a positive association of the age of housing with blood lead concentration (online supplemental table S3), but no other associations with demographic characteristics.

**Table 2:** Lead and mercury exposure levels in pregnant women enrolled in the UK PEAR Study

Toxic metal	Biosample	n	Concentration		
			Mean (SD)	Median (IQR)	Range
Lead	Whole blood (μg/L)	262	5.8 (6.2)	4.6 (3.5, 5.9)	1.7–78.1
	Urine (μg/L)	249	0.50 (0.99)	0.32 (0.18, 0.55)	0.05–10.70
	Urine (μg/g creatinine)	249	0.90 (3.62)	0.44 (0.31, 0.63)	0.03–52.26
Mercury					
Total	Whole blood (μg/L)	262	0.83 (0.64)	0.69 (0.34, 1.19)	0.07–3.40
Speciated	Whole blood (μg/L)	262			
Hg^2+^			0.23 (0.21)	0.14 (0.14, 0.14)	0.14–1.54
MeHg^+^			0.77 (0.64)	0.60 (0.30, 1.08)	0.14–3.81
Total	Urine (μg/L)	249	0.54 (0.44)	0.42 (0.24, 0.74)	0.01–3.43
Total	Urine (μg/g creatinine)	249	0.94 (1.94)	0.65 (0.35, 1.00)	0.03–28.37

MeHg, methylmercury; PEAR, Pregnancy, the Environment And nutRition.

**Figure 3:**
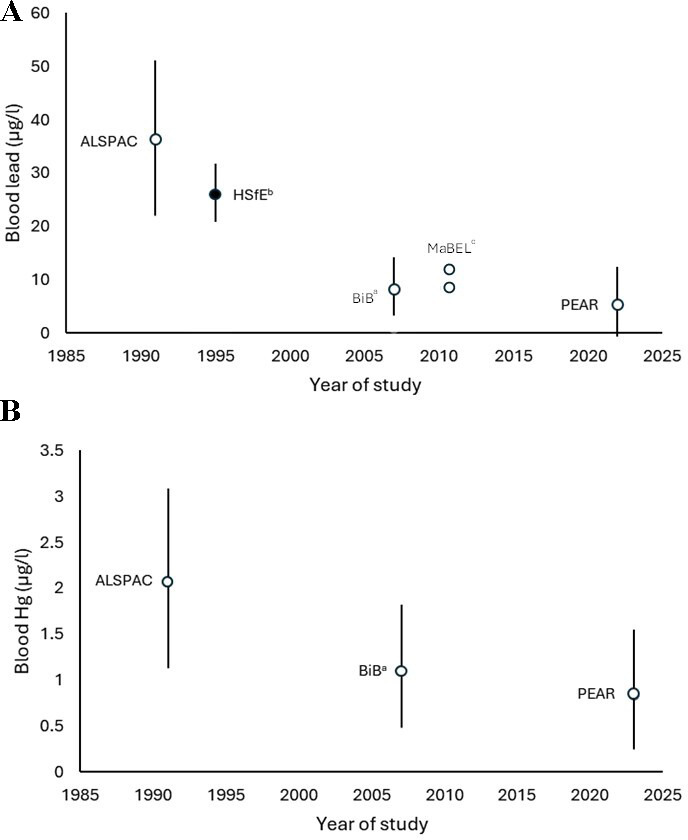
(a) Whole blood lead concentrations in pregnancy in the UK from 1990 onwards. White circles: pregnant women; black circles: women of childbearing age. Mean values with SD except ^a^median value with IQR; ^b^mean with SE; ^c^geometric mean. ALSPAC; Bristol area, n=4285^13^; HSfE (representative sample for England; women age 25–44 years), n=1276^16^,^18^; BiB (Bradford) reported in the HELIX Study, n=126^14^; MaBEL, Leeds, n=98 South Asian and n=38 White^15^; PEAR (Bristol area), n=262. See online supplemental table S4 for full details of data for pregnant women. (**b**) Blood mercury concentrations in pregnancy in the UK from 1990 onwards (no published population-level data for non-pregnant populations). Mean values with SD for total mercury except ^a^median value with IQR. ALSPAC (Bristol area) (n=4134)^13^; BiB (Bradford) reported in the HELIX Study (n=126)^14^; PEAR (Bristol) (n=262). See online supplemental table S6 for full details of data for pregnant women. ALSPAC, Avon Longitudinal Study of Parents and Children; BiB, Born in Bradford; HSfE, Health Survey for England; MaBEL, Mother’s and Baby’s Exposure to Lead; PEAR, Pregnancy, the Environment And nutRition.

**Figure 4:**
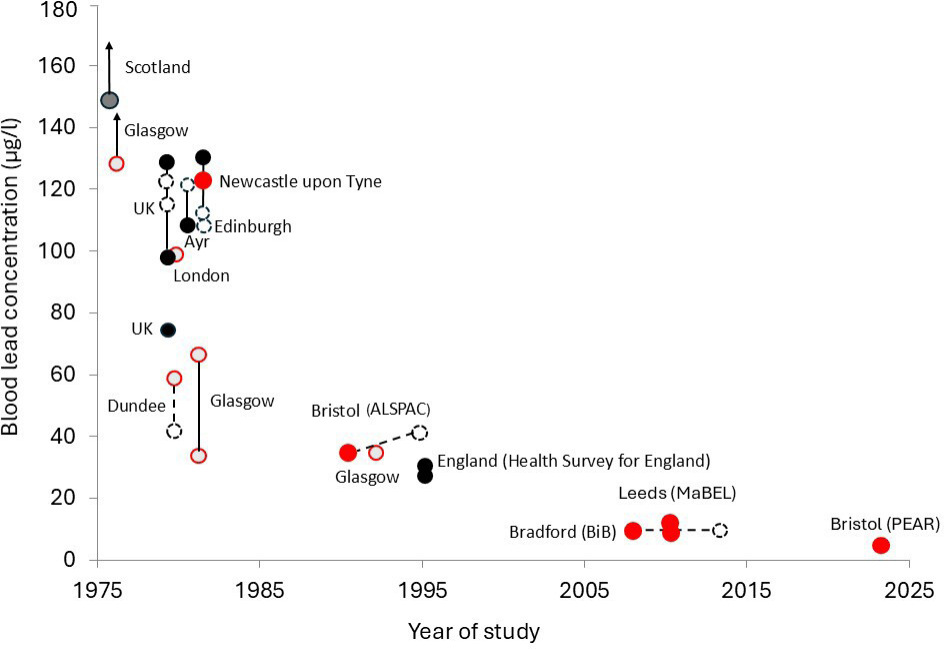
Blood lead concentrations at all life stages in the UK with time. Scotland^64^; Glasgow n=232^65^; UK (n=1617 adults and 1789 children)^66^ ^67^; Ayr (n=31 adults, 13 infants)^68^; Newcastle on Tyne (n=184 pregnant women, 47 non-pregnant women and 23 children)^69^; UK n=7378^70^; London (n=28)^71^; Dundee (n=1165 mother-child pairs)^72^; Glasgow (n=236 postpartum women and baby pairs)^73^; Edinburgh (n=495)^74^; Glasgow (n=342)^75^; Bristol (ALSPAC) (n=4285 pregnant women and 535 children)^13 42 76^; England (Health Survey for England; n=3323 men and 3583 women)^16^,^18^; Bradford (BIB) (n=126) women and children)^14^; Leeds (MaBEL) (n=98 South Asian women and 38 White women)^15^; Bristol (PEAR) (n=262). Red circles, pregnant women; red circle with light grey fill, women at delivery; black circle, adult; dashed line circle, babies/children; dark grey circle, studies in 1970s in Scotland, adults, mean values 145–356 μg/l^65^. Dotted line, mother–child pairs; solid line, data within the same study. ALSPAC, Avon Longitudinal Study of Parents and Children; BiB, Born in Bradford; HSfE, Health Survey for England; MaBEL, Mother’s and Baby’s Exposure to Lead.

The mean blood lead value is 84% lower than that found in ALSPAC in 1991/1992^28^; in comparison with the median value for the BiB study in 2007–2011,^14^ the Pregnancy, the Environment And nutRition (PEAR) median value is 47% lower (figure 3 and online supplemental table S4); and in comparison with MaBEL in 2011, the mean value is 58% lower than geometric mean value in South Asian women and 34% lower than in White women.^15^ Blood lead concentrations in the UK population, including those in pregnancy, have declined in an approximately exponential manner since the 1970s (figure 4). The PEAR Study results are similar to contemporary concentrations found in similar high-income countries (HICs) in Europe and the USA, but lower than those found in low-and-middle income countries (LMICs) (online supplemental table S5). In addition, fewer than 0.5% of the values in PEAR were >50 µg/L (UK level of concern) and fewer than 1% were >35 µg/L (US level of concern) (table 3).

**Table 3:** Summary of national and global levels of concern for pregnant women and women of childbearing age for lead and mercury

	Country	Year	Reference	Population guidance	Level of concern
Lead					
Population	UK	2021	^77^	Public health intervention concentration for pregnant women	≥50 µg/L whole blood (0.48 µmol/L)
	USA*	2021	^78^	Public health intervention concentration for pregnant women	≥35 µg/L whole blood
	WHO	2021	^79^	Public health intervention concentration for pregnant women	≥50 µg/L whole blood (0.48 µmol/L)
	Australia	2016	^80^	Public health intervention concentration for pregnant women	≥50 µg/L whole blood (0.48 µmol/L)
Occupational	UK	2002	Control of lead at work^44^	Trigger for medical surveillance for women of reproductive capacity	≥200 µg/L whole blood (1.92 µmol/L)
	EU	2024	^81^	Trigger for medical surveillance for women of childbearing age: revised limit applicable from 2029	≥45 µg/L whole blood (0.43 µmol/L) for women of childbearing age or ≥national reference levels for population not occupationally exposed if reference level available
Mercury^‡§^					
Population	Germany	2023	^29^	HBM value for general population—derived for women of childbearing age. Also recommended for other population groups	HBM-I: 5 µg/L whole blood† HBM-II: 15 µg/L whole blood†
				HBM for general population	HBM-I: 7 µg/L urine, 4 µg/g Ct† HBM-II: 25 µg/L urine, 20 µg/g Ct†

* See also Ruckart *et al*^82^ for a US state-level documentation of prevention policies and practices.

† HBM-I value is that below which the Commission judge that there is no risk of adverse health effects and consequently no need for action. HBM-II is the value at which adverse effects cannot be excluded with sufficient certainty and intervention is recommended.

‡ There are no levels of concern specifically for pregnant women to our knowledge. WHO (2011) recommends a provisional tolerable weekly intake from foods other than fish and shellfish (inorganic mercury) of 4 µg/kg body weight for adults and children.^83^

§ US healthcare providers reference ranges for total Hg typically 0–5 µg/L whole blood and 20 ng/mL urine; UK hospital trust reference ranges typically 0–16 nmol/L whole blood (0–3.2 µg/L) and 0–2 µmol/mol creatinine; for examples, see New York State Department of Health^84^ and NHS South Tees Hospital,^85^ respectively.

Ct, creatinine; HBM, human biomonitoring value.

Urine lead concentrations were correlated with blood lead concentrations (blood vs urine (n=242) Spearman’s r=0.34 (95% CI 0.22 to 0.45) (p<0.001); blood versus urine standardised for creatinine (n=242) Spearman’s r=0.57 (95% CI 0.48 to 0.65) (p<0.001)).

Blood mercury concentrations (total and speciated; n=262) and urine mercury concentrations (expressed as μg/L and μg/g creatinine; n=249) are shown in table 2 and figure 2. The data were left skewed as was also the case in ALSPAC.^27^ Methylmercury contributed 77% of the total mercury when expressed as measured (Hg^2+^ + MeHg) and 93% when expressed as measured total mercury. There were positive associations of blood total mercury levels with being underweight, not being White, having higher education qualifications, having dental amalgams added or removed during pregnancy and not having a special diet (online supplemental table S3).

The mean total blood mercury value is about 60% lower than in pregnant women in ALSPAC in 1991–1992. In comparison with the median value for BiB in 2007–2011, the PEAR median value is about 37% lower (figure 3 and online supplemental table S6). These results are similar to contemporary concentrations found in similar HIC, but lower than those found in LMIC (online supplemental table S7). There are no national or global levels of concern for mercury specifically for pregnant women for comparison (table 3), although Germany has recommendations for women of childbearing age (Human Biomonitoring Value (HBM-1; value below which it is judged that there is no risk of adverse health effects and consequently no need for action): 5 µg/L whole blood^29^).

Urine total mercury concentrations were correlated with blood total mercury concentrations (blood vs urine (n=242) Spearman’s r=0.19 (95% CI 0.00 to 0.00) (p<0.003); blood versus urine standardised for creatinine (n=242) Spearman’s r=0.15 (95% CI 0.00 to 0.00) (p<0.021)).

## Discussion

We have documented contemporary levels of exposure to lead and mercury in blood and urine in a group of pregnant women in the UK. For both lead and mercury, this represents only the third data point available in pregnancy in the UK since 1990. The data suggest a marked decline in exposure levels for lead and mercury in the UK. However, this is tempered by consensus that there is no lower limit for the adverse effects of toxic metals and that there has been a sequential lowering of levels of concern in HIC including the USA and UK (see table 3). The results are similar to concentrations found in pregnant women in other HIC. The PEAR data make an exceptionally valuable contribution to the understanding of contemporary exposures to toxic metals in a vulnerable group in the UK. Nevertheless, the participation of urban, predominantly white, educated women from affluent households underscores the need for nationally representative biomonitoring efforts.

Toxic metal exposures can be measured in a variety of biological matrices, including blood, urine and hair, and for lead, nails, bone and teeth. In general, blood and urine concentrations represent recent exposure (acute exposure), whereas teeth and bone represent longer-term exposure (chronic exposure) but are more technically difficult and expensive to analyse. Increased bone turnover in pregnancy results in an elevation of blood lead during pregnancy,^30^ so that blood and urine are the most frequently used for monitoring lead exposure in populations, and were moderately correlated in the present study, suggesting that urine lead might have use as a screening tool with venous blood sampling for suspected cases. Nails and hair can be useful for screening, but are prone to external contamination.^31^ For mercury, distinguishing methylmercury from total mercury is informative because of their differing toxicokinetic properties.

### UK trends

Only four studies in the UK have reported on lead exposure in pregnancy since 1990: ALSPAC in 1991–1992, BiB in 2006–2010, MaBEL in 2011 and PEAR in 2023–2024. ALSPAC and PEAR are based in Bristol (south-west England), BiB in Bradford (northern England) and MaBEL in Leeds (northern England), so all four studies are in post-industrial cities that might be expected to have relatively high exposure levels with a legacy of lead mining and processing. All the locations have relatively high levels of lead naturally in soil/rock.^32^ BiB has a high proportion of participants from an ethnic minority background in comparison with ALSPAC and PEAR,^33^,^35^ while MaBEL sought to document the difference between South-Asian and White women: previous work has suggested that ethnic minority background might be associated with increased exposure to lead through the use of traditional medicines and cosmetics.^28^

Government agency interest in population health related to toxic metals in the UK has been low, in contrast to the USA and some other European countries, which have regular population monitoring and extensive public health guidance.^5 36 37^ Children and pregnant women as vulnerable groups in the UK are not subject to monitoring or screening. However, the UK has a history of mining and industrial activity, which can contribute to an environment enriched in toxic metals. Within a European context, the UK has the oldest housing stock overall, with a legacy of coal dust and leaded paint that can be disturbed during renovations. While the sale of leaded paint was banned in the UK in 1992, although with some exceptions, historic sources often remain in situ.^38^ Leaded aviation fuel is still used by piston engine aircraft and is a source of contamination for those living near UK airports^39^ (Bristol has an international airport on the south side of the city, and a historic airport, now housing, in the north of the city). Lead was banned from petrol in the UK in 2000 following a gradual phase out, but continues to be present in the environment.^40^ The chronography of the four UK studies (ALSPAC, BiB, MaBEL, PEAR) encompasses the phase out of leaded petrol in the 1990s and the final ban in 2000. The timeline indicates a corresponding decline in lead concentrations in pregnant women, with a particularly steep decline between 1991/2 (ALSPAC) and 2007/2011 (BiB) and a smaller decline to 2023/2024 (PEAR). An additional sample of women of childbearing age in a Health Survey for England in 1995 provides an intermediate value. The variation in study samples and locations preclude definitive conclusions on the magnitude of the decline, but it is suggestive of a decline in levels over time.

Toxic metal concentrations are typically greater in lower socioeconomic groups, but in ALSPAC, the opposite was found, challenging this common assumption.^41^ The PEAR sample, also in Bristol, was generally a high socioeconomic group, suggesting that the mean level might be different in a more representative group. When additional UK studies including adults and children are also considered (eg, levels in the offspring of the index pregnancies in ALSPAC and BiB),^14 42^ it seems likely that the banning of leaded petrol has been the major influence on the decline in exposure in this timeline, in line with international declines associated with removal of lead from petrol.^43^

In comparing our data with the UK Health Security Agency (UKHSA) level of concern for pregnant women (≥50 µg/L; table 3), there appears superficially to be little evidence for public health policy to be focused on this group, or to support routine population screening. However, the aim must still be to minimise exposure to lead during pregnancy to minimise the risk of preterm birth and other adverse effects on the fetus.^13^ Incongruously, acceptable limits for occupational exposure for women of childbearing age in the UK in Control of Lead at Work regulations are set at a level four times that of the UKHSA level of concern for pregnant women^44^ (the former would be removed from working with significant lead exposure sources if they reached the upper limit) (table 3).

Few population data are available on exposure to mercury in the UK, with only three being in pregnancy (ALSPAC, BiB and PEAR). Similarly to lead, there was a clear decline in mercury exposure from the early 1990s to the present. The present study is the first in the UK to our knowledge to distinguish MeHg from Hg;^2+:^ most of the blood mercury was in the form of MeHg rather than Hg^2+^, suggesting that seafood was a major source of exposure. Inorganic mercury is released naturally into the atmosphere from, for example, volcanoes, forest fires and weathering of mercury-containing rocks; anthropogenic sources include the burning of fossil fuels, especially coal, and recycling of batteries and e-waste. The most toxic form of mercury, methylmercury, is formed from methylation of inorganic mercury in the aquatic environment and it is biomagnified in aquatic food chains. The timeline of the three UK studies covers a period of decline in major industry in the UK, combined with an increase in regulatory processes that may filter mercury out of emissions, including from crematoria (amalgam from dental fillings) and a reduction in the use of dental amalgam in pregnant women (from 2019).^45^ UK government data suggest a decline in mercury emissions from crematoria and industrial sites from 2018 to 2021.^46^ In contrast, however, there has also been a global increase in mercury exposure from battery and e-waste recycling, although this may affect LMIC more than HIC.^47^ Dietary intakes of fish, the primary source of methylmercury, were similar in ALSPAC and PEAR,^21 48^ and this stability is also reflected nationally in data from the National Diet and Nutrition Survey.^49^ It is possible that the decline in total mercury exposure between the two studies reflects a combination of a decline in industrial sources of mercury with a resultant decline in methylmercury generation in biosystems.

### International comparisons

In an international context, PEAR blood lead levels are comparable to those found in recent studies in pregnant women in HIC (mean value 5.8 µg/L, compared with values reported in, eg, Norway 9.1, USA 4.4, Poland 2.0 and Japan 6.3 µg/L (online supplemental table S5). Rich sequential data in the USA has charted the decline in levels in the child and adult population in the National Health and Nutrition Examination Survey (NHANES) from the 1970s onwards, with levels in the adult population declining from geometric mean 16.8 µg/L in 1999 to 8.2 µg/L in 2016,^50 51^ similar to the decline seen in pregnancy in the UK documented here. Similarly, PEAR blood mercury levels are comparable to those in pregnant women in Canada (mean value 0.83 (SD 0.64) vs geometric mean 0.57 µg/L). Higher values in LMIC may reflect, for example, artisan gold-extraction activity.^52^ These comparisons suggest that the UK’s policies to control environmental lead and mercury have had some success in terms of matching exposures with similar countries, but this should not support complacency.

### Guideline comparisons

We found that very few women exceeded UK levels of concern for blood lead (<0.5%), or even the slightly more conservative US level of concern (<1%). However, as previously noted, there is increasing evidence supporting adverse effects at all levels of exposure.^8^ Moreover, while these blood lead levels represent the amount of lead available to the developing fetus, young children are at greater risk for exposures to leaded paint, house dust and water. While a reduction of a few IQ points may not be of concern for the life course of an individual child, at a population level this can result in a significant loss of intellectual capital, with a significant impact on gross domestic product as well as disease burden. For example, Larsen and Sanchez-Triana estimated the total global cost of lead exposure at US$6 trillion in 2019, equivalent to 7% of gross domestic product, of which 23% was the present value of future income losses from IQ loss.^53^ This must drive sustained efforts on policy development and implementation to minimise exposures. There are no blood levels of concern for total mercury specifically for pregnancy for comparisons, although there is guidance in Germany for women of childbearing age.^29^

### Implications for NHS diet guidance and public health information

In the UK, there are two NHS guidelines for pregnant women on foods/drinks to avoid or reduce that are relevant to exposure to toxic metals.^9^ The first is advice to avoid game meats such as goose, partridge or pheasant. This is to avoid ingestion of meat contaminated with lead shot and/or lead shot splinters.^54^ Although game meat is rarely eaten by pregnant women in the UK, those that eat it prepregnancy tend not to avoid it during pregnancy,^22^ so this advice needs to be better targeted and publicised. Following recognition that there is a risk to young children and women of childbearing age who are frequent consumers of lead-shot game meat,^55 56^ lead ammunition will be banned in the UK from 2029, with some exceptions.^57^ The second is advice on avoiding certain types of fish (high level predatory fish such as marlin and swordfish) and limiting oily fish such as salmon and mackerel to reduce mercury exposure: aspects of this advice are not well understood and can result in reduced consumption or avoidance of fish altogether during pregnancy.^21^ There is increasing evidence that the beneficial effect of fish, likely from the nutrients it contains (long-chain fatty acids, iodine, selenium, vitamin D, choline, etc), outweigh any potential adverse effects from mercury.^58 59^ As fish intakes in the UK have not increased,^21 48 49^ but we document a decline in blood mercury levels from the early 1990s until the mid 2020s, we endorse the recommendation that the guidance needs to be simplified and headlined with advice to eat at least two portions of fish per week.^21^

UK central public health information on lead is provided by the UKHSA^60^ and includes advice on mitigation of lead in drinking water, soil and paint, plus advice on lead in some kitchen items, lead residue on clothing, lead contamination of traditional medicines, herbs and spices, and cosmetics, and where to go for individual advice. However, it does not mention other simple strategies to minimise exposure, such as washing hands before eating and drinking, running the tap in older houses before using the water, washing fruit and vegetables before consumption, eating a healthy and varied diet, not wearing shoes indoors, and regular vacuuming and damp-dusting in the home. This advice is not specifically publicised to pregnant women as part of their routine care. Systems for individual blood testing are largely focused on children rather than including pregnant women: the UKHSA currently recommends that clinicians should order a blood lead level test if a child is showing any pica behaviour and should have a low threshold for screening for lead exposure in children with learning disabilities or behavioural disorders.^61^ Population level screening, or screening in pregnancy, for any toxic metal is currently not recommended in the UK,^62^ in contrast with routine testing and population sampling in other countries including the USA.^63^

### Strengths and limitations

This study has several strengths. It provides a valuable contemporary measurement of toxic metal exposures (mercury and lead in the same group of women) in a vulnerable group in the UK, significantly expanding and updating a very small body of data from the UK. All the women were at a similar gestational age (range 8.3–15.9 weeks), largely avoiding variation in the blood volume expansion in the second trimester. ICP-MS is an accurate and sensitive analytical method, allowing for the detection of low-level exposures. We have included data on speciation of blood mercury, which indicates sources of exposure and for which there are no previous UK data for pregnancy. We have also included a standardisation for urine concentrations to account for variations in the level of hydration and of placentation: this should be usual practice to enable comparisons between population samples.

As limitations, our findings are not generalisable to other areas of the UK, where exposures may be different due to, for example, variations in the density of industry and the metallic content of rocks and soil, or in the amount of lead-shot game consumed. Similarly, the results are not generalisable to populations with a different demographic profile (particularly regarding the limited representation of diverse ethnic groups and relatively high socioeconomic status). A high proportion of blood samples had Hg^2+^ <LOQ, although we chose a recognised statistical method to be able to input these values. Some of the categories used for ANOVA analyses contained small case numbers, which weakens confidence in some of the associations.

## Conclusions

This study provides an impetus for the collection of data on exposures to toxic metals among women and other vulnerable populations in the UK, such as children, using larger samples and more representative populations. All the participants had detectable concentrations of blood lead: while few participants in this study had lead exposure levels above the UK limit of concern, any exposure in pregnancy has potential consequences for the fetus. These results also point to the need for continued vigilance in both the prevention and remediation of lead contamination in the environment. There is still a great need for an improved readily accessible central source of public health information: this could include advice on DIY, particularly around dealing with old paint, as well as on domestic cleaning regimens and washing of hands and food items. Adequate monitoring of imported products such as herbs and spices, cosmetics and cookware must be maintained. The mercury exposure levels are reassuring in a worldwide context and support continuing efforts to promote fish-eating in pregnancy (and the wider population) as positively beneficial.^58 59^

## Supplementary material

10.1136/bmjopen-2025-114169 online supplemental file 1

## Data Availability

No data are available.

## References

[1] World Health Organization (2018). International programme on chemical safety: ten chemicals of major public health concern. https://www.who.int/news-room/photo-story/detail/10-chemicals-of-public-health-concern.

[2] Osman K, Akesson A, Berglund M (2000). Toxic and essential elements in placentas of Swedish women. Clin Biochem.

[3] Rudge CV, Röllin HB, Nogueira CM (2009). The placenta as a barrier for toxic and essential elements in paired maternal and cord blood samples of South African delivering women. J Environ Monit.

[4] Mei Z, Liu G, Zhao B (2023). Emerging roles of epigenetics in lead-induced neurotoxicity. Environ Int.

[5] Centers for Disease Control and Prevention (2021). Guidelines for the identification and management of lead exposure in pregnant and lactating women. https://stacks.cdc.gov/view/cdc/147837.

[6] Gould E (2009). Childhood lead poisoning: conservative estimates of the social and economic benefits of lead hazard control. *Environ Health Perspect*.

[7] O’Connor LE, Spill MK, Trivedi R (2025). Mercury exposure and childhood outcomes: an overview of systematic reviews. Environ Res.

[8] Taylor CM, Tilling K, Golding J (2016). Low level lead exposure and pregnancy outcomes in an observational birth cohort study: dose-response relationships. BMC Res Notes.

[9] NHS (2023). Foods to avoid in pregnancy. https://www.nhs.uk/pregnancy/keeping-well/foods-to-avoid/.

[10] EFSA Panel on Contaminants in the Food Chain (CONTAM) (2010). Scientific opinion on lead in food. EFSA J.

[11] Golding J, Steer CD, Hibbeln JR (2013). Dietary predictors of maternal prenatal blood mercury levels in the ALSPAC birth cohort study. *Environ Health Perspect*.

[12] Karagas MR, Punshon T, Sayarath V (2016). Association of rice and rice-product consumption with arsenic exposure early in life. JAMA Pediatr.

[13] Taylor CM, Golding J, Emond AM (2014). Lead, cadmium and mercury levels in pregnancy: the need for international consensus on levels of concern. J Dev Orig Health Dis.

[14] Haug LS, Sakhi AK, Cequier E (2018). In-utero and childhood chemical exposome in six European mother-child cohorts. Environ Int.

[15] Neelotpol S, AWm H, Woolridge MW (2026). Ethno-cultural risk of ante-natal lead exposure among South Asian women in the UK. Soc Sci Med.

[16] Primatesta P, Dong W, Bost L (1998). IEH Report on Recent UK Blood Lead Surveys.

[17] Bost L, Dong W, Primatesta P (1998). The relationship between blood lead and blood pressure in the english population. IEH Report on Recent Blood Lead Surveys.

[18] Bost L, Primatesta P, Dong W (1999). Blood lead and blood pressure: evidence from the Health Survey for England 1995. J Hum Hypertens.

[19] Sakamoto M, Haraguchi K, Tatsuta N (2021). Plasma and red blood cells distribution of total mercury, inorganic mercury, and selenium in maternal and cord blood from a group of Japanese women. Environ Res.

[20] Committee on Toxicity (2025). Discussion paper on the effects of mercury on human health: conclusions. https://cot.food.gov.uk/The%20effects%20of%20mercury%20on%20maternal%20health%20-%20Conclusions.

[21] Beasant L, Ingram J, Taylor CM (2023). Fish consumption during pregnancy in relation to national guidance in England in a mixed-methods study: the PEAR study. Nutrients.

[22] Beasant L, Ingram J, Tonks R (2023). Provision of information by midwives for pregnant women in England on guidance on foods/drinks to avoid or limit. BMC Pregnancy Childbirth.

[23] Harris PA, Taylor R, Thielke R (2009). Research electronic data capture (REDCap)—A metadata-driven methodology and workflow process for providing translational research informatics support. J Biomed Inform.

[24] REDCap (2025). REDCap (Research Electronic Data Capture). https://projectredcap.org/.

[25] (2025). Twilio. https://www.twilio.com/en-us.

[26] Hornung RW, Reed LD (1990). Estimation of average concentration in the presence of nondetectable values. Appl Occup Environ Hyg.

[27] Taylor CM, Kordas K, Golding J (2017). Effects of low-level prenatal lead exposure on child IQ at 4 and 8 years in a UK birth cohort study. Neurotoxicology.

[28] Taylor CM, Golding J, Hibbeln J (2013). Environmental factors predicting blood lead levels in pregnant women in the UK: the ALSPAC study. PLoS One.

[29] Human Biomonitoring Commission (2023). Reference and HBM values. https://www.umweltbundesamt.de/en/topics/health/commissions-working-groups/human-biomonitoring-commission/reference-hbm-values.

[30] Gulson BL, Jameson CW, Mahaffey KR (1997). Pregnancy increases mobilization of lead from maternal skeleton. J Lab Clin Med.

[31] Barbosa F, Tanus-Santos JE, Gerlach RF (2005). A critical review of biomarkers used for monitoring human exposure to lead: advantages, limitations, and future needs. Environ Health Perspect.

[32] UK Soil Observatory (2024). Contaminant distribution in soil. https://mapapps2.bgs.ac.uk/ukso/home.html.

[33] Wright J, Small N, Raynor P (2013). Cohort profile: the Born in Bradford multi-ethnic family cohort study. Int J Epidemiol.

[34] Fraser A, Macdonald-Wallis C, Tilling K (2013). Cohort profile: the Avon Longitudinal Study of Parents and Children: ALSPAC mothers cohort. Int J Epidemiol.

[35] Boyd A, Golding J, Macleod J (2013). Cohort profile: the 'Children of the 90s' - the index offspring of the Avon Longitudinal Study of Parents and Children. Int J Epidemiol.

[36] Becker K, Schroeter-Kermani C, Seiwert M (2013). German health-related environmental monitoring: assessing time trends of the general population’s exposure to heavy metals. Int J Hyg Environ Health.

[37] Etchevers A, Glorennec P, Le Strat Y (2015). Screening for elevated blood lead levels in children: assessment of criteria and a proposal for new ones in France. IJERPH.

[38] Society for the Protection of Ancient Buildings (2025). Lead paint. https://www.spab.org.uk/advice/lead-paint.

[39] Mills A, Peckham S (2022). Lead exposure from general aviation emissions in the UK: a review and call for action. Public Health Chall.

[40] Resongles E, Dietze V, Green DC (2021). Strong evidence for the continued contribution of lead deposited during the 20th century to the atmospheric environment in London of today. Proc Natl Acad Sci U S A.

[41] Vrijheid M, Martinez D, Aguilera I (2012). Socioeconomic status and exposure to multiple environmental pollutants during pregnancy: evidence for environmental inequity?. J Epidemiol Community Health.

[42] Chandramouli K, Steer CD, Ellis M (2009). Effects of early childhood lead exposure on academic performance and behaviour of school age children. *Arch Dis Child*.

[43] Angrand RC, Collins G, Landrigan PJ (2022). Relation of blood lead levels and lead in gasoline: an updated systematic review. Environ Health.

[44] Health and Safety Executive (2002). Control of lead at work (third edition). https://books.hse.gov.uk/gempdf/L132.pdf.

[45] Department of Health & Social Care (2019). National plan to phase down use of dental amalgam in England. https://assets.publishing.service.gov.uk/media/5d12135be5274a0698d0b8a4/phasing-down-use-of-dental-amalgam-in-england.pdf.

[46] Department for Environment, Food and Rural Affairs (2024). H3: emission of mercury and persistent organic pollutants to the environment. https://oifdata.defra.gov.uk/themes/biosecurity-chemical-and-noise/H3/.

[47] World Health Organization (2024). Electronic waste (e-waste). https://www.who.int/news-room/fact-sheets/detail/electronic-waste-%28e-waste%29.

[48] Hibbeln JR, Davis JM, Steer C (2007). Maternal seafood consumption in pregnancy and neurodevelopmental outcomes in childhood (ALSPAC study): an observational cohort study. The Lancet.

[49] Office for Health Improvement & Disparities (2025). National diet and nutrition survey 2019 to 2023. https://www.gov.uk/government/statistics/national-diet-and-nutrition-survey-2019-to-2023/national-diet-and-nutrition-survey-2019-to-2023-report.

[50] Egan KB, Cornwell CR, Courtney JG (2021). Blood lead levels in U.S. children ages 1-11 years, 1976-2016. Environ Health Perspect.

[51] Wang T, Zhou YP, Sun Y (2021). Trends in blood lead levels in the U.S. From 1999 to 2016. Am J Prev Med.

[52] Palacios-Valoyes E, Salas-Moreno MH, Marrugo-Negrete JL (2024). Biomonitoring of mercury and lead levels in the blood of children living near a tropical river impacted by artisanal and small-scale gold mining in Colombia. Toxics.

[53] Larsen B, Sánchez-Triana E (2023). Global health burden and cost of lead exposure in children and adults: a health impact and economic modelling analysis. Lancet Planet Health.

[54] Pain DJ, Cromie RL, Newth J (2010). Potential hazard to human health from exposure to fragments of lead bullets and shot in the tissues of game animals. PLoS One.

[55] (2024). Statement on HSE proposals to restrict the use of lead ammunition in Great Britain.

[56] (2025). Food Standards Agency. Lead-shot game. Food Stand Agency.

[57] (2025). Toxic lead ammunition banned to protect Britain’s countryside. GOVUK.

[58] Golding J, Taylor C, Iles-Caven Y (2022). The benefits of fish intake: results concerning prenatal mercury exposure and child outcomes from the ALSPAC prebirth cohort. Neurotoxicology.

[59] Spiller P, van Wijngaarden E, Adams HR (2023). Net effects explains the benefits to children from maternal fish consumption despite methylmercury in fish. Neurotoxicology.

[60] (2024). UK Health Security Agency. Lead: Information for the public. Lead Inf Public.

[61] (2021). Information on lowering of the lead intervention concentrations for children and pregnant women in England.

[62] (2018). UK National Screening Committee. Screening for elevated blood lead levels in asymptomatic children aged 1 to 5 years.

[63] Jacobs DE, Brown MJ (2023). Childhood lead poisoning 1970-2022: Charting progress and needed reforms. Journal of Public Health Management & Practice.

[64] Moore MR, Goldberg A, Meredith PA (1979). The contribution of drinking water lead to maternal blood lead concentrations. Clin Chim Acta.

[65] Moore MR, Meredith PA, Campbell BC (1977). Contribution of lead in drinking water to blood lead. The Lancet.

[66] (1983). Department of the Environment, Central Directorate on Environmental Pollution. European Community Screening Programme for Lead: United Kingdom Results for 1981.

[67] (1981). Department of the Environment, Central Directorate on Environmental Pollution. European Screening Programme for Lead: United Kingdom Results for 1979-1980.

[68] Sherlock J, Smart G, Forbes GI (1982). Assessment of lead intakes and dose-response for a population in Ayr exposed to a plumbosolvent water supply. Hum Toxicol.

[69] Alexander FW, Delves HT (1981). Blood lead levels during pregnancy. Int Arch Occup Environ Heath.

[70] Pocock SJ, Shaper AG, Walker M (1983). Effects of tap water lead, water hardness, alcohol, and cigarettes on blood lead concentrations. J Epidemiol Community Health.

[71] Kovar IZ, Strehlow CD, Richmond J (1984). Perinatal lead and cadmium burden in a British urban population. Arch Dis Child.

[72] Zarembski PM, Griffiths PD, Walker J (1983). Lead in neonates and mothers. Clin Chim Acta.

[73] Moore MR, Goldberg A, Pocock SJ (1982). Some studies of maternal and infant lead exposure in Glasgow. Scott Med J.

[74] Laxen D, Raab G, Fulton M (1987). Children’s blood lead and exposure to lead in household dust and water — a basis for an environmental standard for lead in dust. Sci Total Environ.

[75] Watt GCM, Britton A, Gilmour WH (1996). Is lead in tap water still a public health problem? An observational study in Glasgow. BMJ.

[76] Taylor CM, Kordas K, Golding J (2017). Data relating to prenatal lead exposure and child IQ at 4 and 8 years old in the Avon Longitudinal Study of Parents and Children. Neurotoxicology.

[77] (2024). UK Health Security Agency. Lead: environmental and public health intervention.

[78] Ruckart PZ, Jones RL, Courtney JG (2021). Update of the blood lead reference value - United States, 2021. MMWR Morb Mortal Wkly Rep.

[79] (2021). World Health Organization. WHO guideline for clinical management of exposure to lead: executive summary.

[80] (2016). National Health And Medical Research Council Australian Government. Managing individual exposure to lead in Australia - a guide for health practitioners.

[81] (2024). Directive (EU) 2024/869 of the European Parliament and of the Council of 13 March 2024 amending Directive 2004/37/EC of the European Parliament and of the Council and Council Directive 98/24/EC as regards the limit values for lead and its inorganic compounds and for diisocyanates.

[82] Ruckart PZ, Schondelmeyer R, Allen A (2024). State-level childhood lead poisoning prevention policies and practices in the United States: 2022-2023. Pediatrics.

[83] World Health Organization (2011). Safety evaluation of certain contaminants in food. WHO food additives series: 63. FAO JECFA monographs 8. https://iris.who.int/bitstream/handle/10665/44520/9789241660631_eng.pdf?sequence=1&isAllowed=y.

[84] New York State Department of Health (2024). Understanding mercury exposure levels. https://www.health.ny.gov/environmental/chemicals/mercury/docs/exposure_levels.htm.

[85] NHS South Tees Hospital (2024). Mercury (blood and urine). https://www.southtees.nhs.uk/services/pathology/tests/mercury-blood-and-urine/?swpmtx=68a78e4ed635915357471c1ee74e7b43&swpmtxnonce=4d8a821a1b.

