# Supplementary material for: Lead and mercury exposure in pregnant women in the UK: a cross-sectional observational study (the PEAR Study)
Source: BMJ Open. 2026 Jun 29;16(6):e114169. doi: 10.1136/bmjopen-2025-114169 (PMC13330889; doi:10.1136/bmjopen-2025-114169)
Supplement: online supplemental file 1 [file bmjopen-16-6-s001.pdf]

Lead and Mercury Exposures in Pregnant Women in the UK: The PEAR Study

Supplementary material

Supplementary Text	Analyses and limits of quantification
Supplementary Table 1	Quality assurance for analyses
Supplementary Table 2	Limits of quantification (LOQ)
Supplementary Table 3	Demographic characteristics of PEAR Study participants by lead and mercury levels
Supplementary Table 4	Lead exposure levels in pregnant women enrolled in the UK PEAR Study and comparison with other UK studies (µg/l)
Supplementary Table 5	Examples of other findings for lead exposure in pregnancy from HIC, UMIC and LMIC countries (published 2020 to present): whole blood lead (maternal/cord)
Supplementary Table 6	Mercury exposure levels in pregnant women enrolled in the UK PEAR Study and comparison with other UK studies
Supplementary Table 7	Examples of other findings for mercury exposure in pregnancy from HIC, UMIC and LMIC countries (published 2020 to present): whole blood mercury (maternal/cord)
Supplementary references	

Supplementary text

Analytical methods

Blood lead and mercury (total mercury)

Lead and total mercury were analysed in the same analysis. The blood samples were diluted 1 in 100 in an alkaline diluent (0.1% m/v ethylenediaminetetraacetic acid EDTA (Fisher Scientific, Loughborough, UK), 1% v/v ammonia (Super pure, Romil, Cambridge, UK), 0.5% v/v sodium dodecyl sulphate (10% solution, ultrapure, Life Technologies, NY, USA), 1 µg/L platinum ICPMS standard as the internal standard, and 10 mg/L gold ICPMS (all ICPMS standards were Aristar ICPMS standards, VWR, Lutterworth UK) and analysed by ICPMS using an Thermo ICap RQ ICPMS (Hemel Hempstead, UK).

Standards (lead and mercury, also diluted from Aristar 1000 mg/L ICPMS standards, VWR, Lutterworth UK) were matrix matched with equine horse blood (TCS BioSciences, Buckingham UK) and calibrated in the ranges of 0.03-3 µg/L for lead and 0.01-1 µg/L for mercury. To ensure quality within the analysis commercially available quality controls samples used analysed in every analysis, ClinChek (RECIPE Chemicals, Munich, Germany). Participation in external quality schemes was also undertaken G-EQAS https://app.g-equas.de/web/

Blood mercury speciation (Hg^{2+} and $MeHg^{+}$)

Blood samples were diluted 1 in 20 following an extraction process. 50 µL whole blood was added to 0.75 ml extract solution (0.1% v/v HCl (Ultrapure, Romil, Cambridge UK), 0.1% v/v methanol and 0.05% m/v L-cysteine) in a capped tube. After vortexing, the tube was placed in an ultrasonic water bath for 30 min at 30oC. Then 50 µL methanol was added and the tubes were centrifuged for 10 min at 3 500 rps. 500 µL blood extract was pipetted into a Nanosep centrifugal filter (300k, Cytiva, via VWR, Lutterworth, UK) and this was centrifuged for 10 min at 13 500 rps. The filtrate was then analysed by LC-ICPMS. Matrix matched blood standards were prepared 0.1-1 µg/L from a mixed standard of Hg2+ (1 000 mg/L ICPMS standard, VWR, Lutterworth, UK) and MeHgCl (1 mg/L solution from Brooks Rand, Seattle, USA) using the same extraction method.

Quality control samples used included a certified reference material SRM 955d (stored at -80°C until prepared), Clinchek blood QC level 1 and 2 (RECIPE Chemicals, Munich, Germany) as well as prepared blood sample spiked with 1 µg/L of each species, stored at -20°C.

Analysis was achieved using liquid chromatography coupled to ICPMS (LC-ICPMS) using an 8900 ICPMSMS in single quadrupole no gas mode to measure 202Hg. The ICPMS used a Duramist high performance inert nebuliser (Glass Expansion Pocasset, MA, USA), was a nebuliser gas flow of 1.01 L/min and sample depth of 10 mm. The LC system was a 1290 Agilent LC Infinity II consisting of a 15 cm column Acclaim 120 C18 5µm (Thermo Fisher) at 20oC. The isocratic mobile phase (5% v/v methanol, 0.4% m/w L-cysteine, 0.06 mol/L ammonium acetate, 0.05% v/v 2-mercaptoethanol pH 6.7-7(all from Fisher Scientific, Loughborough, UK) had a flow rate of 1 ml/min. 100 µL sample was injected. Chromatography settings used Gaussian smoothing and Agile 2 peak integration in Mass Hunter (Agilent Technologies, Cheadle, UK).

Urine lead

Urine samples were diluted 1 in 20 with 1% v/v nitric acid (Fisher Chemicals, Loughborough, UK) with 0.2% m/v EDTA and 10 µg/L platinum (ICP-MS Aristar grade 1000 mg/L standard, VWR, Lutterworth UK) added as an internal standard. Calibration standards 0.01-10 µg/L made from a multi-elemental ICPMS standard (Spex CertiPrep, Metuchen, NJ, USA). The autosampler wash solution was 1% v/v nitric acid.

The samples were analysed in normal mode using a Thermo ICap RQ ICP-MS (Hemel Hempstead, UK). To ensure quality within the analysis commercially available quality controls samples used analysed in every analysis using Lypochek ClinChek (Recipe, Munich Germany) urine quality control samples. Participation in external quality schemes was also undertaken G-EQAS https://app.g-equas.de/web/

Urine mercury

Urine samples were diluted 1 in 20 with 1% v/v nitric acid (Fisher Chemicals, Loughborough, UK) with 0.2% m/v EDTA and 10 µg/L platinum (ICP-MS Aristar grade 1000 mg/L standard, VWR, Lutterworth UK) added as an internal standard and 1 mg/L gold (ICP-MS Aristar grade 10 000 mg/L standard, VWR, Lutterworth UK) added to stabilise the mercury in solution.

Calibration standards 0.05-1 µg/L were made fresh for each analysis from a ICPMS mercury standard (ICPMS Aristar grade 1000 mg/L standard, VWR, Lutterworth UK) with 1% v/v nitric acid and 1 mg/L gold added to the standards. The autosampler wash solution was 1% v/v nitric acid with 1 mg/L gold.

The samples were analysed in normal mode using a combination of Thermo ICap Q and Icap RQ ICP-MS (Hemel Hempstead, UK). To ensure quality within the analysis commercially available quality controls samples used analysed in every analysis using Bio-Rad urine quality control samples (Hemel Hempstead, UK). Participation in external quality schemes was also undertaken G-EQAS https://app.g-equas.de/web/

Creatinine measurements

All urine results were reported as creatinine corrected. Creatinine was determined by an automated alkaline picrate method, using an ABX Pentra 400 spectrophotometer (HORIBA ABX UK, Northampton, UK). An internal QC material made from a pooled urine sample and stored frozen in 1 mL aliquots was used. The QC sample was thawed at room temperature before use and analysed after each calibration. All QC results fell within the acceptable range. Participation in external quality assurance schemes for creatinine measurements was also undertaken in a UK scheme (RIQAS organised by Randox Laboratories Limited, Belfast, N. Ireland).

Quality assurance

External quality assurance was available and attained for all analytes except for the blood mercury species, methyl and inorganic mercury were none was available. All analysis was undertaken, and results reported with commercially available QC materials measured in each analysis with results within the stated ranges as shown in Supplementary Table 1.

Supplementary Table 1 Quality control assurance for ICP-MS analyses

QC material	Element/species, LOQ of method (mean concentration ± SD)		Certified level
**Blood: Total lead and total mercury (μg/L)**	**Lead**	**Mercury (total)**	
LOQ[a]	0.2	0.1	
ClinChek Level 1 blood Lot 2193	34.89 ± 1.03	3.16 ± 0.15	Pb 27.2–40.7<br>Hg 2.05–3.81
ClinChek Level 2 blood Lot 2193	91.02 ± 2.46	8.72 ± 0.33	Pb 27.2–40.7<br>Hg 6.55–10.9
**Urine: Total lead (μg/L)**	Lead		
LOQ [a]	0.07		
ClinChek Level 1 Lot 2334	13.29 ± 0.14		Pb 10.6-15.8
ClinChek Level 2 Lot 2334	27.71 ± 0.51		Pb 22.2-33.4
Biorad Level 1 Lot 69251	48.11 ± 1.38		Pb 41.0-61.5
**Blood: Mercury species (μg/L)**	**Hg^{2+}**	**$MeHg^{+}$**	
LOQ [a]	0.2	0.2	
ClinChek Level 1 blood Lot 2193	1.92 ± 0.19	0.48 ± 0.06	Total Hg 2.05–13.81
ClinChek Level 2 blood Lot 2193	1.92 ± 0.19	0.48 ± 0.06	Total Hg 2.05–13.81
IQC @ 1 μg/L	1.12 ± 0.11	1.08 ± 0.09	
SRM 1 955d (n=3)	0.29 ± 0.05 (72.8%)	0.59 ± 0.02 (94.6%)	
SRM 2 955d (n=3)	2.23 ± 0.93 (104.6%)	3.93 ± 0.23 (102.3 %)	
**Urine: Total mercury (μg/L)**			
LOQ*	0.02		
Biorad Level 1 urine Lot 69241	31.4 ± 2.0		Hg 28.4–42.7

[a] LOQ in undiluted biological matrix

Supplementary Table 2 Limits of quantification (LOQ) for ICP-MS analyses

	**LOQ (µg/l)**	**Replace by LOQ/√2**	**n <LOQ**
Blood Pb	0.200	0.200/1.414 = 0.141	0
Blood Hg	0.100	0.100/1.414 = 0.070	22
Hg^{2+}	0.200	0.200/1.414 = 0.141	210
MeHg	0.200	0.200/1.414 = 0.141	11
Urine total Hg	0.02	0.02/1.414 = 0.014	1
Urine total Pb	0.07	0.07/1.414 = 0.050	16

Supplementary Table 3 Demographic characteristics of PEAR study participants by blood lead and mercury levels

Characteristic	n	Blood lead		Blood total mercury	
		**Mean (SD)**	**P value**	**Mean (SD)**	**P value**
Age (years)					
<25	7	4.29 (0.38)	0.686	0.41 (0.30)	0.686
25-35	144	6.14 (7.66)		0.84 (0.69)	
>35	77	5.61 (4.21)		0.82 (0.51)	
BMI classification [a]					
Underweight (<18.5 kg/m^2)	8	5.92 (4.76)	0.135	1.25 (0.57)	**0.019**
Normal weight (18.5-24.9 kg/m^2)	68	7.97 (11.23)		0.87 (0.61)	
Overweight/Obese (≥25 kg/m^2)	56	4.98 (1.95)		0.68 (0.54)	
Parity					
0	124	5.89 (4.92)	0.999	0.81 (0.60)	0.800
≥1	105	5.89 (8.08)		0.83 (0.66)	
Gestational age at sampling (weeks)					
<12	70	5.22 (1.97)	0.511	0.82 (0.72)	0.990
12-14	173	6.14 (7.44)		0.83 (0.61)	
>14	4	4.10 (1.79)		0.80 (0.41)	
Ethnicity					
White	200	5.64 (6.63)	0.104	0.78 (0.61)	**0.008**
Black/African/Caribbean/Black British/Asian/Asian British/Mixed/multiple ethnic groups	28	7.79 (5.74)		1.11 (0.62)	
Household income					
<£20,000	11	4.48 (0.94)	0.304	0.50 (0.47)	0.185
£20,000 to <£50,000	62	7.01 (10.91)		0.81 (0.69)	
≥£50,000	148	5.66 (4.02)		0.86 (0.61)	
Don't know					
Highest educational qualification					
None/GCSE/Vocational level 1 and 2/AS or A level/Vocational level 3	53	4.86 (2.65)	0.184	0.67 (0.59)	**0.048**
University degree (BSc, BA, MA)/professional qualification/Vocational levels 4 and 5/University higher degree (MSc, PhD)	176	6.22 (7.31)		0.86 (0.63)	
Smoker (tobacco)					

Characteristic	n	Blood lead		Blood total mercury	
		Mean (SD)	P value	Mean (SD)	P value
No, not at all	227	5.92 (6.58)	0.739	0.83 (0.63)	0.383
Yes, less often than every day/Yes, every day	3	4.65 (0.54)		0.51 (0.39)	
Vaping[a]					
No, not at all	179	6.11 (7.28)	0.540	0.81 (0.61)	0.162
Yes, less often than every day/Yes, every day	4	3.88 (1.60)		0.38 (0.38)	
Type of housing					
Detached house (2 storeys or more)/Detached house (bungalow)	42	4.99 (1.99)	0.067	0.83 (0.62)	0.067
Semi-detached house (2 storeys or more)/Semi-detached house (bungalow)	95	5.30 (3.69)		0.88 (0.68)	
Terraced house/townhouse (2 storeys or more)	59	7.87 (11.45)		0.87 (0.60)	
Flat or apartment/Maisonette or duplex/Other	31	5.34 (3.74)		0.55 (0.45)	
Age of housing					
Before 1963	88	6.87 (6.13)	**0.029**	0.83 (0.67)	0.911
From 1962 up to 1992	52	4.86 (2.92)		0.85 (0.59)	
From 1992	59	4.42 (2.34)		0.75 (0.61)	
Dental amalgam fillings added/removed during this pregnancy					
No	205	6.02 (6.89)	0.545	0.78 (0.58)	**0.039**
Yes	3	3.60 (1.37)		1.51 (1.65)	
Special diet					
No	196	5.73 (6.32)	0.233	0.85 (0.63)	**0.048**
Yes	31	7.25 (7.98)		0.61 (0.58)	

Analysis of variance (ANOVA).

Supplementary Table 4 Lead exposure levels in pregnant women enrolled in the UK PEAR Study and comparison with other UK studies (µg/l)

Publication	Study	Year of study	Location (UK)	Biosample	n	Mean (SD)	Median (IQR)	Range
Present	PEAR	2023–2024	Bristol	Maternal whole blood	262	5.8 (6.2)	4.6 (3.5, 5.9)	1.7–78.1
Neelotpol et al. (2026)[1]	MaBEL	2011	Leeds	Maternal whole blood	98 South Asian and 38 White	South Asian: GM 13.34<br>White: GM 8.84	-	-
Haug et al. (2018)[2]	BiB (HELIX Study)	2007-2010	Bradford	Maternal whole blood	126	-	8.68 (6.19, 12.10)	Maximum 51.50
Primatesta et al. (1998), Bost et al. (1998), Bost et al. (1999) [3–5]a	HSfE	1995	England	Women of child-bearing age (16-24 years old), whole blood	321	27 (SE 2), GM 21	-	-
				Women of child-bearing age (25-45 years old), whole blood	1276	26 (SE 5), GM 23	-	-
Taylor et al. (2014)[6]	ALSPAC	1991–1992	Bristol	Maternal whole blood	4285	36.7 (14.7)	34.1	2.9-191.4
Alexander & Delves (1981)[7]	-	Not reported	Ashington and Newcastle	Maternal whole blood	165	8 weeks: 132.7<br>Term: 105.7<br>Mean: 122.4 (31.1)	-	-

UK public health intervention concentration for pregnant women (UK Health Security Agency[8]): 50.0 µg/l (0.24 µmol/l).

[a]Values are for adult women of child-bearing age rather than pregnant.

Study acronyms: ALSPAC, Avon Longitudinal Study of Parents and Children; BiB, Born in Bradford; HSfE, Health Survey for England; MaBEL, Mother's and Baby's Exposure to Lead; PEAR, Pregnancy, the Environment And nutRition.

Supplementary Table 5 Examples of other findings for lead exposure in pregnancy from HIC, UMIC and LMIC countries (published 2020 to present): whole blood lead (maternal/cord)

	Study	Publication	Year of study	Location	Biosample	n	Mean (SD)	Median (IQR)	Range
HIC	NICHD National Children's Study Vanguard Studies	Stanek et al. (2023)[9]	2019-2014	USA	Maternal whole blood (µg/l)	426	GM 4.4		
HIC		Tabassum et al. (2023)[10]	NR	Saudi Arabia	Maternal serum (µg/l)	30	3.865 (SD 0.020) (control)		
						30	7.796 (SD 0.551) (recurrent pregnancy loss)		
HIC	-	Gajewska et al. (2021)[11]	2018-2020	Poland	Maternal whole blood (µg/l)			18.0 (9.0, 27.0) (read from graph in Fig 2b for healthy pregnant women)	4.0-52.0
HIC	**-**	Kot et al. (2021)[12]	2014-2015	Poland	Maternal whole blood (µg/l)	136	2.0 (1.0)	1.0	0.0-14.0
					Cord whole blood (µg/l)	136	2.0 (0.0)	2.0	1.0-6.0
HIC	JECS	Goto et al. (2021)[13]	2011-2014	Japan	Maternal whole blood (µg/l)	20,000	6.3 (0.3)	6.3 (28, 35)	1.6-74
HIC	PROTECT	Ashrap et al. (2020)[14]	2011-2017	Puerto Rico	Maternal whole blood (µg/l)	1183	GM 3.3 (GSD 1.6) Median	3.3 (2.5, 4.3)	
HIC	MIREC	Polevoy et al. (2020)[15]	2008-2011	Canada	Late pregnancy	427	GM 12.7 (GSD 1.3)	12.7 (10.0-16.8)	
HIC	MOCEH	Shah-Kulkarni et al. (2020)[16], Lee et al. (2020)[17], Kim et al. (2020)[18]	2006-2010	Republic of Korea	Maternal whole blood (µg/l)				
					Early pregnancy	523	GM 13.3 (GSD 15.1)	13.4 (10.1-17.3)	
					Late pregnancy	427	GM 12.7 (GSD 1.3)	12.7 (10.0-16.8)	
					Cord whole blood (µg/l)	321	GM 9.2 (GSD 15.9)	9.4 (7.1-12.3)	
HIC	MoBa	Weyde et al. (2021)[19]	2003-2007	Norway	Maternal whole blood (µg/l)	652	9.1 (9.0)	7.9 (6.1, 10.5)	2.2-160.0
UMIC	-	Waeyeng et al.	2021	Thailand	Maternal whole blood	80	46.8 (15.5) (42.5%		

	Study	Publication	Year of study	Location	Biosample	n	Mean (SD)	Median (IQR)	Range
		(2022)[20]			(μg/l)		≥50)		
UMIC	-	Lu et al. (2022)[21]	2020-2021	China	Maternal whole blood (μg/l)	195	7.61 (SD 2.64)	7.24 (5.83, 8.91)	
UMIC	-	Vigeh et al. (2021)[22]	2016-	Iran	Maternal whole blood (μg/l)	166	49.6 (66.77)		5.17-709.82
UMIC	-	Ou et al. (2020)[23]	2016-2018	China	Maternal whole blood (μg/l)	150	27.17 (spontaneous abortion)		
UMIC	Shahrekord Cohort study	Tousizadeh et al. (2023)[24]	2015-	Iran	Maternal whole blood (mg/l)[d]	120	2.01 (2.47)		
UMIC	PROGESS	Niedzwiecki et al. (2021)[25]	2007-2011	Mexico	Early pregnancy	523	GM 13.3 (GSD 15.1)	13.4 (10.1-17.3)	
LMIC	-	Sahu et al. (2024)[26]	NR	India	Maternal whole blood (μg/l)	150	17.28 (control)		
					Cord whole blood (μg/l)	90			0.1-93.2 (n=5 ≥50)

HIC, High Income country, UMIC, Upper-Middle Income country; LMIC, Lower-Middle Income country; LIC, Low Income country (World Bank Classifications by income level 2024-5).

[a]Women of child-bearing age.

[b]Representative sample.

[c]Part of Europe-wide HELIX Study.

[d]We attempted to contact the corresponding author to verify the unit but were unfortunately unable to do so.

GM, geometric mean; GSD, geometric standard deviation.

Study acronyms: JECS, Japan Environment and Children's Study; HSfE, Health Survey for England; MIREC, Maternal-Infant Research on Environmental Chemicals; MoBa, Mother, Father and Child Cohort Study; MOCEH, Mother and Children Environmental Health; NICHD, National Institute of Child Health and Human Development; PROGRESS, Programming Research in Obesity, Growth, Environment, and Social Stressors; PROTECT, Puerto Rico Testsite for Exploring Contamination Threats.

Supplementary Table 6 Mercury exposure levels in pregnant women enrolled in the UK PEAR Study and comparison with other UK studies

Study	Publication	Year of study	Location	Biosample	n	Mean (SD)	Median (IQR)	Range
PEAR	Present	2023-2024	Bristol	Maternal whole blood (µg/l)	262			
				Total		0.83 (0.64)	0.69 (0.34, 1.19)	0.07-3.40
				Speciated				
				Hg^{2+}		0.23 (0.21)	0.14 (0.14, 0.14)	0.14-1.54
				$MeHg^{2+}$		0.77 (0.64)	0.60 (0.30, 1.08)	0.14-3.81
				Maternal urine (µg/l)	249	0.54 (0.44)	0.42 (0.24-0.74)	0.01-3.43
				Maternal urine (µg/g Ct)	249	0.94 (1.94)	0.65 (0.35-1.00)	0.03-28.37
BiB (HELIX Study)	Haug et al (2018)[2]	2007-2010	Bradford	Maternal whole blood (µg/l)	126	-	1.10 (0.72, 1.79)	Maximum 4.94
ALSPAC	Taylor et al. (2014)[6]	1991-1992	Bristol	Maternal whole blood (µg/l)	4134	2.07 (1.10)	1.86	0.17-12.76

UK public health intervention concentration for pregnant women: no guideline.

Ct, creatinine.

Study acronyms: ALSPAC, Avon Longitudinal Study of Parents and Children; BiB, Born in Bradford; PEAR, Pregnancy, the Environment And nutRition.

Supplementary Table 7 Examples of other findings for mercury exposure in pregnancy from HIC, UMIC and LMIC countries (published 2020 to present): whole blood mercury (maternal/cord)

	Study	Publication	Year of study	Location	Biosample	n	Mean (SD)	Median (IQR)	Range
HIC	PROTECT	Ashrap et al. (2020)[14]	2011-2017	Puerto Rico	Maternal whole blood (μg/l)	1183	GM 1.20 (GSD 1.70)	1.20 (0.85, 1.70)	
HIC	MIREC	Polevoy et al. (2020)[15]	2008-2011	Canada	Maternal whole blood (μg/l)	423 (included imputation): First trimester	*GM 0.568*	*0.602*	*0.045-7.823*
						407 (included imputation): Second trimester	*GM 0.461*	*0.501*	*0.030-18.454*
					Cord whole blood (μg/l)	346 (included imputation)	*GM 0.692*	*0.722*	*0.056-8.826*
HIC	MOHEC	Shah-Kulkarni et al. (2020)[16]	2006-2010	Republic of Korea	Whole blood (μg/l)				
					Early pregnancy	523	GM 3.30 (GSD 1.61)	3.34 (2.44, 4.44)	
					Late pregnancy	427	GM 3.13 (GSD 1.73)	3.13 (2.25, 4.26)	
					Cord blood	321	GM 5.21 (GSM 1.63)	5.33 (3.97, 7.13)	

HIC, High Income country, UMIC, Upper-Middle Income country; LMIC, Lower-Middle Income country; LIC, Low Income country (World Bank Classifications by income level 2024-5).
Values in italics are converted from source data in alternative units.

Study acronyms: MIREC, Maternal-Infant Research on Environmental Chemicals; MOCEH, Mother and Children Environmental Health; PROTECT, Puerto Rico Testsite for Exploring Contamination Threats.

Supplementary references

1. Neelotpol S, Hay AWm & Woolridge MW (2026) Ethno-cultural risk of ante-natal lead exposure among South Asian women in the UK. *Soc. Sci. Med.* **393**, 119034.

2. Haug LS, Sakhi AK, Cequier E, et al. (2018) In-utero and childhood chemical exposome in six European mother-child cohorts. *Env. Int* **121**, 751–763.

3. Primatesta P, Dong W, Bost L, et al. (1998) *Survey of blood lead levels in the population in England, 1995*. 9–34. Norwich, UK: Medical Research Council, Institute for Environmental Health.

4. Bost L, Dong W, Primatesta P, et al. (1998) *The relationship between blood lead and blood pressure in the English population. IEH Report on Recent Blood Lead Surveys.* 85–100.

5. Bost L, Primatesta P, Dong W, et al. (1999) Blood lead and blood pressure: evidence from the Health Survey for England 1995. *J Hum Hypertens* **13**, 123–8.

6. Taylor CM, Golding J & Emond AM (2014) Lead, cadmium and mercury levels in pregnancy: the need for international consensus on levels of concern. *J Dev Orig Health Dis* **5**, 16–30.

7. Alexander FW & Delves HT (1981) Blood lead levels during pregnancy. *Int Arch Occup Env. Health* **48**, 35–9.

8. UK Health Security Agency (2024) Lead: environmental and public health intervention. https://www.gov.uk/government/publications/lead-poisoning-advice-for-the-public-and-healthcare-professionals/lead-environmental-and-public-health-intervention (accessed March 2025).

9. Stanek LW, Grokhowsky N, George BJ, et al. (2023) Assessing lead exposure in U.S. pregnant women using biological and residential measurements. *Sci. Total Environ.*, 167135.

10. Tabassum H, Alrashed M, Malik A, et al. (2023) A unique investigation of thallium, tellurium, osmium, and other heavy metals in recurrent pregnancy loss: A novel approach. *Int J Gynaecol Obstet* **160**, 790–796.

11. Gajewska K, Laskowska M, Almeida A, et al. (2021) Lead Levels in Non-Occupationally Exposed Women with Preeclampsia. *Molecules* **26**.

12. Kot K, Lanocha-Arendarczyk N, Kupnicka P, et al. (2021) Selected Metal Concentration in Maternal and Cord Blood. *Int J Env. Res Public Health* **18**.

13. Goto Y, Mandai M, Nakayama T, et al. (2021) Association of prenatal maternal blood lead levels with birth outcomes in the Japan Environment and Children's Study (JECS): a nationwide birth cohort study. *Int J Epidemiol* **50**, 156–164.

14. Ashrap P, Watkins DJ, Mukherjee B, et al. (2020) Predictors of urinary and blood Metal(loid) concentrations among pregnant women in Northern Puerto Rico. *Env. Res* **183**, 109178.

15. Polevoy C, Arbuckle TE, Oulhote Y, et al. (2020) Prenatal exposure to legacy contaminants and visual acuity in Canadian infants: a maternal-infant research on environmental chemicals study (MIREC-ID). *Env. Health* **19**, 14.

16. Shah-Kulkarni S, Lee S, Jeong KS, et al. (2020) Prenatal exposure to mixtures of heavy metals and neurodevelopment in infants at 6 months. *Env. Res* **182**, 109122.

17. Lee S, Hong YC, Park H, et al. (2020) Combined effects of multiple prenatal exposure to pollutants on birth weight: The Mothers and Children's Environmental Health (MOCEH) study. *Env. Res* **181**, 108832.

18. Kim B, Shah S, Park HS, et al. (2020) Adverse effects of prenatal mercury exposure on neurodevelopment during the first 3 years of life modified by early growth velocity and prenatal maternal folate level. *Env. Res* **191**, 109909.

19. Weyde KVF, Olsen AK, Duale N, et al. (2021) Gestational blood levels of toxic metal and essential element mixtures and associations with global DNA methylation in pregnant women and their infants. *Sci. Total Environ.* **787**, 147621.

20. Waeyeng D, Khamphaya T, Pouyfung P, et al. (2022) Blood Lead Levels among Non-Occupationally Exposed Pregnant Women in Southern Thailand. *Toxics* **10**.

21. Lu Y, Zhang Y, Guan Q, et al. (2022) Exposure to multiple trace elements and miscarriage during early pregnancy: A mixtures approach. *Env. Int* **162**, 107161.

22. Vigeh M, Yunesian M, Matsukawa T, et al. (2021) Prenatal blood levels of some toxic metals and the risk of spontaneous abortion. *J Env. Health Sci Eng* **19**, 357–363.

23. Ou J, Peng P, Qiu L, et al. (2020) Effect of Lead Exposure on Spontaneous Abortion: a Case-Control Study. *Clin Lab* **66**.

24. Tousizadeh S, Mohammadi-Moghadam F, Sadeghi R, et al. (2023) Investigation of the levels of essential and non-essential metals in women with and without abortion history: A study based on the Persian population of the Shahrekord cohort. *Chemosphere* **329**, 138434.

25. Niedzwiecki MM, Eggers S, Joshi A, et al. (2021) Lead exposure and serum metabolite profiles in pregnant women in Mexico City. *Env. Health* **20**, 125.

26. Sahu MC, Upadhyay K, Gupta S, et al. (2024) DNA damage and ALAD polymorphism in high blood lead (Pb) levels of pregnant women attending a tertiary care teaching hospital. *Eur J Obstet Gynecol Reprod Biol X* **22**, 100300.
