# Supplementary material for: Lead and mercury exposure in pregnant women in the UK: a cross-sectional observational study (the PEAR Study)
Source: BMJ Open. 2026 Jun 29;16(6):e114169. doi: 10.1136/bmjopen-2025-114169 (PMC13330889; doi:10.1136/bmjopen-2025-114169)
Supplement: Supplementary file 2 [file bmjopen-2025-114169.reviewer_comments.pdf]

PEER REVIEW HISTORY

BMJ Open publishes all reviews undertaken for accepted manuscripts. Reviewers are asked to complete a checklist review form and are provided with free text boxes to elaborate on their assessment. These free text comments are reproduced below.

ARTICLE DETAILS

Title (Provisional)

Lead and mercury exposure in pregnant women in the UK: a cross-sectional observational study (The PEAR Study)

Authors

Taylor, Caroline M; Mottram, Linda; Ingram, Jenny; Entwistle, Jane; Kordas, Katarzyna; Lanphear, Bruce; Golding, Jean; Morton, Jackie

---

VERSION 1 - REVIEW

**Reviewer**	**1**
**Name**	**Sakuragi, Toshihide**
**Affiliation**	**University of Occupational and Environmental Health Japan, Obstetrics & genecology**
**Date**	**31-Jan-2026**
**COI**	**None**

This is a highly important study evaluating lead and mercury exposure among pregnant women in the UK, with clear relevance to public health policy. The manuscript provides valuable insights into the current status of toxic metal exposure in pregnant women, as well as historical and international comparisons, and is therefore of great interest.

However, I believe that some revisions are needed regarding the presentation and clarity of the tables and figures. Please consider the following comments.

Table 1

•The handling of the BMI category 25–30 is not described. Please clarify and correct this.

•The percentage of gestational age among participants who provided urine samples appears to be incorrect. Please re-check these values.

•The gestational age for participants who provided blood samples is reported as “118.” This may be a typographical error and intended to be “11.8.” Please confirm and correct if necessary.

Figure 1

•Given the relatively small sample size of the present study, simple comparisons without indicating sample size may lead readers to misinterpret the weight of the findings. Please include the sample size (N) for each referenced study.

•Open and closed circles are used in Figure 1; however, their meaning is not immediately clear. Please clarify what these symbols represent, either directly within the figure or in the figure legend, to improve readability.

Figure 2

•Multiple symbol types are used, making the figure difficult to interpret. Although this manuscript focuses on toxic metal exposure among pregnant women, this figure appears to include data from non-pregnant adults and children as well.

•If inclusion of these additional populations is essential, the figure should be revised to improve clarity and readability, or alternatively simplified to focus on pregnant women.

Figure 3

•As with Figure 1, please include the sample size (N) for each referenced study.

•It is unclear which form of mercury is being compared. Please clearly specify whether the data represent total mercury, organic mercury, inorganic mercury, or a combination thereof.

•Given the similarity in the comparison framework, please consider presenting Figure 3 alongside Figure 1, or combining them into a single figure if feasible.

Supplementary Figure 1

•This figure provides highly useful information for understanding the overall study design and results. Please consider presenting it as a main figure rather than as supplementary material.

•If there are restrictions on the number of figures, combining Figure 1 and Figure 3 may be a reasonable option to accommodate this change.

Reviewer 2

Name Hindori-Mohangoo, Ashna D

Affiliation Foundation for Perinatal Interventions and Research in Suriname

Date 15-Mar-2026

COI None

Since one of the objectives of the study is to document metal concentrations over time in pregnant women in the UK, the authors could consider adding lead and mercury values to the results section, lines 238-240 for lead, and lines 258-260 for mercury.

**Reviewer**	**3**
**Name**	**Udumyan, Ruzan**
**Affiliation**	**Örebro Universitet, Clinical Epidemiology and Biostatistics, School of Medical Sciences**
**Date**	**09-Apr-2026**
**COI**	**None**

Review for the manuscript "Lead and Mercury Exposure in Pregnant Women in the UK: The PEAR Study" submitted to BMJ Open

Thank you for the opportunity to review this manuscript. This study describes lead and mercury exposures in a sample of pregnant women in the UK, and compares the findings with previous findings in the UK and other countries.

Some comments are provided below.

Abstract:

RE "Participants   Women ≥18 years old and ≥11 weeks pregnant." could you provide the range of gestational age? According to the strengths and limitations section, women were in the first trimester, so "≥11 weeks" looks misleading.

RE Conclusion: "However, no safe levels have been identified for adverse effects on cognition, preterm birth rate or coronary heart disease prevalence." This conclusion seem to be based upon prior literature- the results section does not suggest that you have examined this? Can you modify accordingly?

Methods:

RE the outcomes definition: would suggest renaming "Analytical methods" to for example "Analytical methods for outcomes assessment" and make relevant changes.

RE "Demographic characteristics were analysed for those who completed the questionnaire and who also provided a urine sample, and correspondingly for those that completed the questionnaire and provided a blood sample."

-Please write instead "Demographic characteristics were described...".

-While reading the "Biosamples" section, I thought both blood and urine samples were collected for each woman /or at least was planed so/. Please clarify.

Results:

How many women provided both urine and blood samples? Are women who completed T1 different from women who completed only T0? Was there a link between socio-demographic variables and lead/mercury levels?

Can you spell out MeHg and Hg2+, e.g. methylmercury (MeHg) at their first appearance?

RE comparison with previous findings: if the populations are comparable and methods are similar (measurement techniques, study design), you could test whether your sample mean is significantly different from the published mean using two-sample comparison (easy to do in Stata: ttesti n1 mean1 sd1 n2 mean2 sd2) if the data are approximately normally distributed.

However, your data does not look normally distributed and might be heavily right-skewed, based on the descriptive statistics. Therefore, you may use a nonparametric one-sample median test (sign test) for example. Was the distribution approximately normal in the ALSPAC study? You may consider including a figure showing the distribution of lead and mercury (e.g. histogram or box-plot).

VERSION 1 - AUTHOR RESPONSE

Comment	Response
**REVIEWER 1**	
Table 1<br>•The handling of the BMI category 25–30 is not described. Please clarify and correct this.	Thank you for spotting this error. The Overweight/Obese category has been corrected to ≥25 kg/m^2.
•The percentage of gestational age among participants who provided urine samples appears to be incorrect. Please re-check these values.	Thank you for spotting this error. The percentages have been corrected. We have checked the whole table on this point and made some minor corrections.
•The gestational age for participants who provided blood samples is reported as "118." This may be a typographical error and intended to be "11.8." Please confirm and correct if necessary.	Thank you for noticing this error. There was a missing decimal point. As noted above, we have checked the whole table and made some minor corrections.
Figure 1<br>•Given the relatively small sample size of the present study, simple comparisons without indicating sample size may lead readers to misinterpret the weight of the findings. Please include the sample size (N) for each referenced study.	This information has been added to the figure legend (now Figure 3) as requested.
•Open and closed circles are used in Figure 1; however, their meaning is not immediately clear. Please clarify what these symbols represent, either directly within the figure or in the figure legend, to improve readability.	The figure legend (now Figure 3) contains the text to explain the symbols as follows. We hope this provides clarity.<br><br>White circles: pregnant women; black circles: women of child-bearing age
Figure 2<br>•Multiple symbol types are used, making	Various symbol types were used to allow the reader to distinguish which

the figure difficult to interpret. Although this manuscript focuses on toxic metal exposure among pregnant women, this figure appears to include data from non-pregnant adults and children as well.	population group the data were based on (now Figure 4). The main message was to illustrate the approximately exponential decline of lead levels in the UK since the 1970s (text to this effect has been added at ln 265) and to provide a UK context for the PEAR data, which was one of our aims. To simplify the figure, the sample numbers have been removed from the figure and added to the figure legend, analogous to Figure 3. We have also noted that the Journal allows colour figures at no extra cost, so we have substituted some coloured symbols and updated the figure legend accordingly. We hope these changes improve the clarity of the figure.
•If inclusion of these additional populations is essential, the figure should be revised to improve clarity and readability, or alternatively simplified to focus on pregnant women.	The figure has been simplified and clarified as described above. The focus on pregnant women is illustrated in Figure 3, but the purpose of Figure 4 is to illustrate the results in the context of the wider UK data as part of our aims.
Figure 3<br>•As with Figure 1, please include the sample size (N) for each referenced study.	This information has been added to the figure legend as requested.
•It is unclear which form of mercury is being compared. Please clearly specify whether the data represent total mercury, organic mercury, inorganic mercury, or a combination thereof.	The values are all for Total mercury. This has been added to the figure legend.
•Given the similarity in the comparison framework, please consider presenting Figure 3 alongside Figure 1, or combining them into a single figure if feasible.	We have combined Figs 1 and 3 into Figure 3 a and b as suggested, and amended the figure legend accordingly.
Supplementary Figure 1<br>•This figure provides highly useful information for understanding the overall study design and results. Please consider presenting it as a main figure rather than as supplementary material.	Thank you for this suggestion. The figure has now been included in the in the main paper as Figure 1.
•If there are restrictions on the number of figures, combining Figure 1 and Figure 3 may be a reasonable option to accommodate this change.	See response above. The limit is 8 figures + tables, so we are within this limit.

**REVIEWER 2**	
Since one of the objectives of the study is to document metal concentrations over time in pregnant women in the UK, the authors could consider adding lead and mercury values to the results section, lines 238-240 for lead, and lines 258-260 for mercury.	The values for lead and mercury are included in Table 2. To avoid repetition of the values shown in the Table, we have not included them in the text, but have referenced the table (ln 255). Similarly for mercury. We hope this is acceptable.
**REVIEWER 3**	
Abstract:<br>RE "Participants Women ≥18 years old and ≥11 weeks pregnant." could you provide the range of gestational age? According to the strengths and limitations section, women were in the first trimester, so "≥11 weeks" looks misleading.	Participants were eligible for recruitment as being ≥11 weeks by date of LMP (recruitment took place before the scan clinic). The gestational age range by scan differed slightly from this. The GA by scan has been added to the abstract (ln 82) and Results (ln 247). We agree the text was misleading, so we have changed 'first trimester' in the Strengths section to '(range 8.3-15.9 weeks), largely avoiding ...' (ln 459).<br><br>Gestation by scan at time of blood test 8.3-15.9 weeks<br>Gestation by scan at time of urine test 8.6-17.5 weeks<br>Gestation by scan at T1 completion 8.0-16.6 weeks
RE Conclusion: "However, no safe levels have been identified for adverse effects on cognition, preterm birth rate or coronary heart disease prevalence." This conclusion seem to be based upon prior literature- the results section does not suggest that you have examined this? Can you modify accordingly?	Thank you for this point. We have added the word 'previously' to the sentence in the Conclusion (ln 90) to indicate that this statement is not based on data in the present paper.
Methods:<br>RE the outcomes definition: would suggest renaming "Analytical methods" to for example "Analytical methods for outcomes assessment" and make relevant changes.	Thank you for this suggestion. We have amended the heading to read 'Analytical methods for blood and urine analyses' for clarity. (ln 222)
RE "Demographic characteristics were analysed for those who completed the questionnaire and who also provided a urine sample, and correspondingly for	This change has been made as requested (ln 232).

those that completed the questionnaire and provided a blood sample."<br><br>-Please write instead "Demographic characteristics were described…".	
-While reading the "Biosamples" section, I thought both blood and urine samples were collected for each woman /or at least was planed so/. Please clarify.	We are sorry this was unclear. The word 'and' has been added (ln 197) to clarify.
Results:<br>How many women provided both urine and blood samples?	We have also added this information to the legend for Fig 1 (flow chart). It is also now stated in the text (ln 270 and ln 290).
Are women who completed T1 different from women who completed only T0? .	Thank you for this suggestion. Unfortunately, we are not able to answer this with our data, as we do not have any data on the characteristics of the participants who only completed T0: this preliminary questionnaire confirmed eligibility criteria and contact details only. This is stated in the methods (ln 180) and we have modified the text to make this clearer.
Was there a link between socio-demographic variables and lead/mercury levels?	Thank you for this suggestion. We have added an additional table (Supplementary Table 3) with these data and referred to it in the Results (ln 257 and ln 280). For lead, the only association was with age of housing (presumably due to the presence of leaded paint and other historic sources of lead). For mercury, there were positive associations of blood total mercury levels with being underweight, not being White, having higher education qualifications, having dental amalgams added or removed during pregnancy and not having a special diet (ln 278). We have noted in the Limitations that some of the categories had small case numbers which weakens confidence in some of these associations (ln 472).
Can you spell out MeHg and Hg2+, e.g. methylmercury (MeHg) at their first appearance?	MeHg has been defined at first mention (ln 152). Hg^{2+} is standard chemical notation and we hope it will be readily understood.

RE comparison with previous findings: if the populations are comparable and methods are similar (measurement techniques, study design), you could test whether your sample mean is significantly different from the published mean using two-sample comparison (easy to do in Stata: ttesti n1 mean1 sd1 n2 mean2 sd2) if the data are approximately normally distributed.<br>However, your data does not look normally distributed and might be heavily right-skewed, based on the descriptive statistics. Therefore, you may use a nonparametric one-sample median test (sign test) for example. Was the distribution approximately normal in the ALSPAC study? You may consider including a figure showing the distribution of lead and mercury (e.g. histogram or box-plot).	Thank you for these welcome suggestions.<br><br>The data are not normally distributed but left-skewed (as in ALSPAC, see Figure 1 in Taylor et al. (2017) Neurotoxicology 62;162-169).<br><br>We have added an extra figure for PEAR data (Figure 2) to illustrate the skew with a histogram. The values for skew and kurtosis are shown in the figure legend and referred to in the Results (ln 255 and 263).<br><br>We agree that it would be useful to test the difference between ALSPAC and PEAR as you suggest. However, one of the assumptions of the Sign test is that it should include 'related groups' indicating that the same participants are present in both groups, which would not be the case here. We would also need to apply for data access permission from ALSPAC, which would take some time and incur a data access fee. Unfortunately, we do not think this is feasible at the moment, but it is certainly something we will take on board for future investigations.

VERSION 2 - REVIEW

Reviewer 1

Name Sakuragi, Toshihide

Affiliation University of Occupational and Environmental Health Japan, Obstetrics & genecology

Date 30-Apr-2026

COI

I have confirmed that appropriate revisions and changes have been made in response to each reviewer's comments. I believe the manuscript is suitable for acceptance.

Reviewer 3

Name Udumyan, Ruzan

Affiliation Örebro Universitet, Clinical Epidemiology and Biostatistics, School of Medical Sciences

Date 08-May-2026

COI

I would like to thank the authors for their responses to previous comments.

Have a minor comment:

In the abstract: "The aims of this study are to: (1) quantify lead and mercury exposures in a sample of pregnant women in the UK;"

I think it is worth adding "in Bristol, UK".
